# Major depressive disorder and current psychological distress moderate the effect of polygenic risk for obesity on body mass index

**DOI:** 10.1038/tp.2015.83

**Published:** 2015-06-30

**Authors:** T-K Clarke, L S Hall, A M Fernandez-Pujals, D J MacIntyre, P Thomson, C Hayward, B H Smith, S Padmanabhan, L J Hocking, I J Deary, D J Porteous, A M McIntosh

**Affiliations:** 1 Division of Psychiatry, University of Edinburgh, Royal Edinburgh Hospital, Edinburgh, UK; 2 Centre for Genomics and Experimental Medicine, Institute of Genetics and Molecular Medicine, University of Edinburgh, Western General Hospital, Edinburgh, UK; 3 MRC Human Genetics, MRC Institute of Genetics and Molecular Medicine, University of Edinburgh, Edinburgh, UK; 4 Centre for Cognitive Ageing and Cognitive Epidemiology, University of Edinburgh, Edinburgh, UK; 5 Population Health Sciences, University of Dundee, Dundee, UK; 6 Institute of Cardiovascular and Medical Sciences, University of Glasgow, Glasgow, UK; 7 Division of Applied Medicine, Institute of Medical Sciences, University of Aberdeen, Aberdeen, UK; 8 Department of Psychology, University of Edinburgh, Edinburgh, UK

## Abstract

Major depressive disorder (MDD) and obesity are frequently co-morbid and this correlation is partly due to genetic factors. Although specific genetic risk variants are associated with body mass index (BMI) and with larger effect sizes in depressed individuals, the genetic overlap and interaction with depression has not been addressed using whole-genome data. Polygenic profile scores for MDD and BMI were created in 13 921 members of Generation Scotland: the Scottish Family Health Study and tested for their association with BMI, MDD, neuroticism and scores on the General Health Questionnaire (GHQ) (current psychological distress). The association between BMI polygenic profile scores and BMI was tested fitting GHQ, neuroticism or MDD status as an interaction term to test for a moderating effect of mood disorder. BMI polygenic profile scores were not associated with lifetime MDD status or neuroticism although a significant positive association with GHQ scores was found (*P*=0.0001, *β*=0.034, *r*^2^=0.001). Polygenic risk for MDD was not associated with BMI. A significant interaction between BMI polygenic profile scores and MDD (*P*=0.0003, *β*=0.064), GHQ (*P*=0.0005, *β*=0.027) and neuroticism (*P*=0.003, *β*=0.023) was found when BMI was the dependent variable. The effect of BMI-increasing alleles was greater in those with MDD, high neuroticism or current psychological distress. MDD, neuroticism and current psychological distress amplify the effect of BMI polygenic profile scores on BMI. Depressed individuals with a greater polygenic load for obesity are at greater risk of becoming obese than control individuals.

## Introduction

Major depressive disorder (MDD) is the second-leading cause of disability worldwide.^1^ Part of this disability may be attributed to physical co-morbidities that are common to MDD, such as being overweight or obese.^2^ Longitudinal studies show baseline obesity increases risk for MDD (odds ratio=1.55, 95% confidence interval=1.22–1.98) and that that MDD increases the odds for developing obesity (odds ratio=1.58; 95% confidence interval=1.33–1.87).^3^ One large epidemiological study found that increased risk for obesity is confined to atypical depression, a subtype of MDD characterized by increased appetite and hypersomnia.^4^ Other studies have found an increased risk of past-month MDD among obese females or individuals with severe obesity (body mass index (BMI) ⩾40).^5^ The reciprocal relationship between obesity and certain subtypes of MDD is not well understood. MDD may arise as a consequence of health problems that accompany obesity or increased appetite may be a symptom of mood disorders.

Obesity and MDD appear to share a common genetic architecture, that is, genetic variants that increase risk for obesity also associate with MDD. Genetic variants in the fat-mass- and obesity-associated FTO gene are associated with MDD. A single-nucleotide polymorphism (SNP) in FTO (rs9939609) that explains 0.34% of the variance in obesity^6^ was found to confer protection against MDD in 6561 depression cases and 21 932 controls.^7^ However, the rs9939609 A allele was found to increase risk for MDD in an independent sample of 1544 cases and 2806 controls, although after adjustment for BMI this was no longer significant. A significant association was detected between rs9939609 and the atypical subtype of MDD in this sample.^8^ MDD has been shown to amplify the effect of obesity-related genetic variants on BMI. An analysis of 88 SNPs in the FTO gene in two independent samples comprising 3734 MDD cases and 1499 controls found consistent evidence that MDD moderates the effect of FTO risk variants on BMI.^9^

The genetic overlap between MDD and obesity/BMI is likely to extend beyond the FTO gene. Variation in BMI has a genetic basis with heritability estimates in the range of 40–70%.^10, 11^ A large genome-wide association study (GWAS) of BMI found 32 loci to be associated at a genome-wide significant level. These 32 loci were found to explain 1.45% of the phenotypic variance in BMI, which is consistent with a polygenic inheritance pattern.^6^ The heritability of MDD has been estimated to be 37%^12^ with 21% of the variance explained by common genetic factors, also suggesting polygenic disease architecture.^13^ Twin studies in female twin pairs have estimated that 12% of the genetic component of depression is shared with obesity.^14^

The aim of this study was to assess whether BMI and MDD have an overlapping polygenic architecture using polygenic profile scores.^15^ This was explored in a large population-based cohort: Generation Scotland: the Scottish Family Health Study (GS:SFHS).^16, 17^ As MDD has been shown to moderate the effect of FTO variants on BMI, we hypothesized that the association between BMI polygenic profile scores and BMI would be moderated by the presence of MDD. We also tested whether this extended to current psychological distress or neuroticism by using scores on the General Health Questionnaire (GHQ-28)^18^ and the Eysenck personality questionnaire for neuroticism,^19^ as these traits are heritable and genetically correlated with MDD in this sample (rG neuroticism=0.58, rG GHQ=0.7).^20^ Furthermore, there is a strong association between neuroticism and depression, and longitudinal studies have found that high premorbid neuroticism is a risk factor for depression.^21, 22^

## Materials and methods

### Sample

#### Generation Scotland: The Scottish Family Health Study

GS:SFHS is a family-based population cohort recruited at random from general practitioners' practices throughout Scotland; the protocol for recruitment is described in detail elsewhere.^16, 17^ The full cohort consists of 23 690 individuals who were over 18 years of age at the time of recruitment and 21 516 of these attended the research clinic. The present study includes 13 921 individuals for whom genome-wide genotype data were available. Demographic information on these individuals is provided in Table 1. MDD was diagnosed using the structured clinical interview for the Diagnostic and Statistical Manual of Mental Disorders (SCID).^23^ A brief screening questionnaire was administered to participants to screen for MDD. Participants were asked “Have you ever seen anybody for emotional or psychiatric problems?” and “Was there ever a time when you, or someone else, thought you should see someone because of the way you were feeling or acting?” If they answered yes to either of these questions (21.7% screened positive), they were asked to complete the SCID.^19^ If they answered no to both of these questions, they were assigned control status. Answers to the SCID provided information on the presence or absence of a lifetime history of MDD, age of onset and number of depressive episodes. Those who completed the SCID but did not meet the criteria for MDD were also defined as controls.

Individuals with a diagnosis of bipolar disorder were removed from this study. The GHQ (GHQ-28)^18^ was completed by 13 715 of the genotyped individuals providing a measure of current psychological distress. The GHQ-28 consists of four subscales designed to assess: (A) somatic symptoms, (B) anxiety and insomnia, (C) social dysfunction and (D) ‘severe depression'. The Eysenck personality questionnaire was completed by 13 838 of the genotyped individuals providing a measure of neuroticism. BMI was calculated using height (cm) and weight (kg) measured by trained clinical staff and was available for 13 827 individuals. All components of GS:SFHS have received ethical approval from the NHS Tayside Committee on Medical Research Ethics (REC Reference Number: 05/S1401/89). Written consent for the use of data was obtained from all participants.

#### Genotyping and polygenic profiling

Blood samples were obtained using standard operating procedures and subsequently stored at the Wellcome Trust Clinical Research Facility Genetics Core (www.wtcrf.ed.ac.uk). Samples were genotyped using the Illumina HumanOmniExpressExome-8v1.0 BeadChip and Infinum chemistry^24^ and processed using the IlluminaGenomeStudio Analysis software v2011.1 (Illumina, San Diego, CA, USA). The details of blood collection and DNA extraction are provided elsewhere.^17^

Quality control on raw genotypes removed individuals with an overall genotyping rate of <99%, SNPs with a minor allele frequency <1%, call rate <99% or a significant deviation from the Hardy–Weinberg equilibrium (*P*⩾1 × 10^−6^) in founder individuals. Multidimensional scaling components were created according to the ENIGMA 1000 genomes protocol^25^ in the software package PLINK.^26^ Four GS:SFHS population outliers were removed and four multidimensional scaling components were used to correct for population stratification in the remaining individuals.

Polygenic profile scores were created in PLINK^26^ according to previously described protocols.^15^ Prior to creating scores, all strand-ambiguous SNPs were removed from the GS:SFHS data set and SNPs were linkage disequilibrium pruned using clump-based pruning (*r*^2^=0.25, 300 kb window). Polygenic profile scores for BMI were calculated using summary data from a GWAS of BMI in 123 865 individuals.^6^ Each BMI-increasing allele was ascribed a weight of ‘1', as the effect sizes for the SNP–BMI associations are not publically available. Polygenic profile scores for MDD were created using summary data from the largest available GWAS of MDD to date comprising 9240 MDD cases and 9519 controls.^27^ Dudbridge estimated that for a trait with a heritability of 44%, 1978 cases and 1978 controls in a training set would provide 80% power to detect association with the same trait in an independent replication sample.^28^ As both the MDD and BMI GWAS were substantially larger than this, we believe we have sufficient power to detect an association between polygenic profile scores and BMI/MDD in the current study. Five polygenic profile scores were created using SNPs associated with MDD or BMI with *P*-value thresholds of 0.01, 0.05, 0.1, 0.5 and 1.

### Statistical analysis

BMI, GHQ and neuroticism were transformed towards normality using the Box–Cox transformation procedure implemented in the MASS package in R.^29^ Continuous variables were scaled to have a mean of 0 and a s.d. of 1, such that the reported regression coefficients (beta) are standardized. Mixed linear models implemented in the ASReml-R (www.vsni.co.uk/software/asreml) software package were used to test the association between polygenic profile scores and BMI, GHQ, neuroticism and MDD. Age, sex, four multidimensional scaling ancestry components and polygenic profile scores were fixed effects. GS:SFHS is a family-based study and to control for relatedness, family structure was fitted as a random effect by creating an inverse relationship matrix using pedigree kinship information. Wald's conditional F-test was used to calculate the significance of fixed effects. To test the interaction of BMI polygenic profile scores with depression status/GHQ/neuroticism, an interaction term was added to the model with BMI polygenic profile scores and depression status/GHQ/neuroticism also included as main effects. The *P*-values presented are raw *P*-values uncorrected for multiple testing. False discovery rate was implemented in the R package ‘fdrtools' to estimate the local false discovery rate. The false discovery rate threshold for statistical significance was estimated to be 0.009.

## Results

The number of individuals in the current study with a lifetime diagnosis of MDD was 2030, and 11 836 individuals were identified as controls. In total, 431 individuals had a diagnosis of current depression at the time of interview. Demographic information is provided in Table 1. MDD cases were significantly younger, had significantly higher GHQ scores, were more likely to be female, had significantly higher neuroticism scores and had larger BMIs than controls. Polygenic risk scores for BMI and MDD were available for 13 921 members of GS:SFHS. The Pearson's correlation between the BMI and MDD polygenic profile scores in 6418 randomly selected unrelated individuals at the *P*⩽0.1 threshold was low and non-significant (cor=0.002, *P*=0.8). Unrelated individuals were used for estimating correlations between polygenic profile scores to avoid confounding due to genetic similarity between family members.

### BMI polygenic profile scores

BMI polygenic profile scores were significantly associated with BMI in GS:SFHS at all *P*-value thresholds (Table 2). Individuals carrying more BMI-increasing alleles had a significantly higher BMI. The polygenic profile score, which explained most of the phenotypic variance in BMI, was at the *P*<0.01 threshold (*β*=0.2, *P*=1.3 × 10^−^^118^) where 4.2% of variance was explained. BMI polygenic profile scores were not significantly associated with MDD or neuroticism (Table 2); however, an association with GHQ scores was observed at all *P*-value thresholds. Individuals scoring higher on the GHQ were found to carry more BMI-increasing alleles. The variance in GHQ explained by the BMI polygenic profile scores was much lower than for BMI (0.1%) ((*P*⩽0.01) *β*=0.03, *P*=0.0001) (Table 2). The association between GHQ scores and BMI polygenic profile scores was reanalysed after controlling for BMI to determine whether the association could be explained by overweight and obese individuals experiencing more psychological distress. At the *P*-value threshold ⩽0.01, there was a ~33% reduction in the effect size but a nominally significant association remained (*β*=0.02, *P*=0.019, *r*^2^=0.0004).

### MDD polygenic profile scores

MDD profile scores were significantly associated with a lifetime history of MDD in GS:SFHS for four of the five *P*-value thresholds, and the greatest amount of variance was explained using a *P*-value threshold of *P*⩽0.1 (*β*=0.01, *P*=0.0001, *r*^2^=0.001) (Supplementary Table 1). The only MDD polygenic profile score associated with BMI was the score containing SNPs associated with MDD at a *P*-value threshold ⩽0.01 (*β*=0.02, *P*e=0.011, *r*^2^=0.0004), but this was not significant after correction for multiple testing. Robust associations between MDD polygenic profile scores and GHQ were found at each *P*-value threshold, and the score explaining the greatest amount of variance was at the *P*⩽0.05 threshold (*β*=0.04, *P*=9 × 10^−^^6^, *r*^2^=0.002). At this threshold, individuals carrying more MDD risk alleles had more current psychological distress measured using the GHQ (Supplementary Table 1). Positive associations between MDD polygenic profile scores and neuroticism were also found at each *P*-value threshold, and the greatest amount of variance explained was at the *P*⩽1 threshold (*β*=0.04, *P*=0.0001, *r*^2^=0.001). Individuals who have higher neuroticism scores carry more MDD risk alleles.

The association between polygenic risk for MDD and BMI was tested in MDD cases only (*N*=2030). No significant associations were found (data not shown).

### Interaction between BMI polygenic profile score and MDD/GHQ/neuroticism

**Figure 1:**
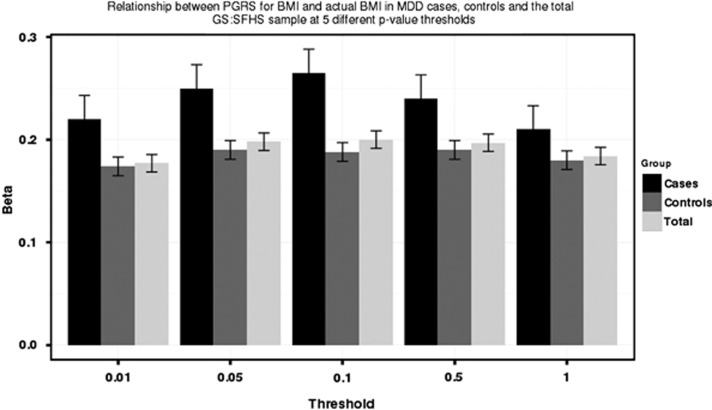
Relationship between BMI polygenic profile scores and measured BMI in Generation Scotland: the Scottish Family Health Study. Bars represent the total sample, cases or controls at each polygenic profile score *P*-value threshold cutoff. *The y* axis represents the standardized beta for the association between BMI polygenic profile score and BMI, error bars represent s.e. BMI, body mass index; GS:SFHS, Generation Scotland: the Scottish Family Health Study; MDD, major depressive disorder; PGRS, polygenic risk score.

**Figure 2:**
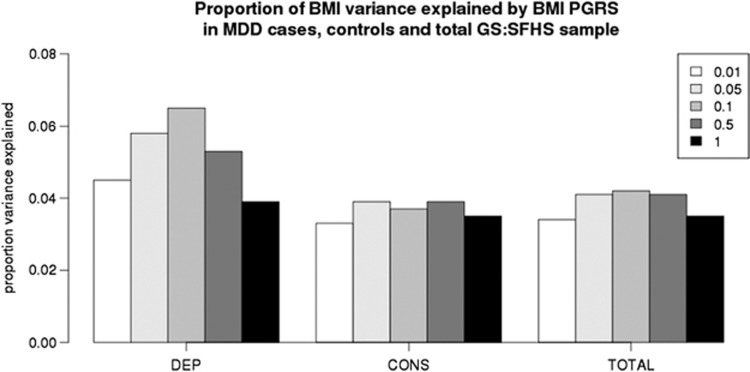
Proportion of variance in BMI explained by BMI polygenic profile scores at five different thresholds in GS:SFHS (14 k). DEP=2030 MDD cases, CONS=11 836 controls, TOTAL=13 921 of total sample. All associations significant at *P*⩽3.2 × 10^−19^. BMI, body mass index; DEP, major depressive disorder; GS:SFHS, Generation Scotland: the Scottish Family Health Study; PGRS, polygenic risk score.

A significant interaction between BMI polygenic profile scores and MDD status was found at the *P*-value threshold ⩽0.1 in relation to BMI (*β*=0.064, *P*=0.0032) (Table 3). Figure 1 illustrates the relationship between BMI polygenic profile scores and BMI in MDD cases and controls. The effect of BMI polygenic profile scores on BMI is greater in individuals with MDD (MDD cases *β*=0.265 vs controls *β*=0.188). Figure 2 shows the amount of variance explained by BMI polygenic profile scores in MDD cases, controls and the total sample. At a *P*-value threshold of *P* ⩽0.1, BMI polygenic profile scores explain 6.5% of the variance in BMI among MDD cases in comparison with 3.7% of the variance in controls. The interaction term was not significant at the *P*-value thresholds other than *P* ⩽0.1, but the direction of effect was consistent across all five scores.

A significant interaction between BMI polygenic profile scores and GHQ was also observed in relation to BMI, and this was significant for all five polygenic profile scores (Table 3). The greatest effect was seen using all SNPs (*P*⩽1) (*β*=0.027, *P*=0.0005). The interaction-term effect was in the same direction as for MDD: the effect of carrying more BMI-increasing alleles on BMI is greater in individuals experiencing greater levels of current psychological distress. To demonstrate this, the relationship between BMI and BMI polygenic profile score was tested in each quartile of the GHQ score (Supplementary Figure 1). Similarly, a significant interaction between BMI polygenic profile scores and neuroticism scores was found when BMI was the dependent variable. The largest effect size for the interaction was observed at the *P*-value threshold of *P*⩽0.1 (*β*=0.023, *P*=0.003).

To test whether the effect of MDD on BMI polygenic risk was due to current depression/mood rather than lifetime MDD status, an interaction between current depression (*N*=431) and polygenic risk for BMI was tested (Table 3). Nominally significant associations were observed at two from five *P*-value thresholds, but these did not withstand correction for multiple testing.

## Discussion

There is little evidence for genetic overlap between BMI and MDD in this large population-based cohort using a polygenic profile score approach. BMI polygenic profile scores for BMI were not associated with a lifetime history of MDD in GS:SFHS, and polygenic risk for MDD was not associated with BMI. BMI polygenic profile scores and GHQ scores were positively associated, suggesting some genetic overlap between obesity and current psychological distress. MDD status was found to amplify the effect of BMI polygenic profile scores on BMI. BMI-increasing alleles have a stronger effect on BMI in depressed individuals. A similar relationship was observed with GHQ and neuroticism. GHQ and neuroticism scores were found to moderate the effect of BMI polygenic profile scores on BMI.

Previous studies have found genetic variants in the FTO gene to be associated with MDD.^7, 8^ Using an aggregate score of all genetic variants associated with BMI, we find no association with MDD status. The genetic overlap between MDD and BMI may be restricted to the FTO gene, or as suggested by Milaneschi *et al.*,^8^ BMI risk variants may only be relevant for atypical depression. We do find modest genetic overlap between BMI and GHQ scores, as individuals with higher GHQ scores were found to carry more BMI-increasing alleles. One explanation for this is that BMI-increasing genetic variants increase risk for current psychological distress but not lifetime MDD. Alternatively, there may be genetic overlap between BMI and MDD, but we have greater power to detect an association with GHQ scores due to the trait being continuous versus binary. High GHQ scores have previously been found to be associated with obesity,^30, 31^ and it has been suggested that obesity increases the risk for common mental disorders as indexed by the GHQ.^32^ We also find no association between BMI polygenic profile scores and neuroticism, further suggesting that it is current psychological distress (measured using the GHQ) rather than a tendency to lower mood that overlaps with polygenic risk for BMI.

A recent study of SNPs across the FTO gene found that depressive status moderated the association with BMI in 10 of the SNPs studied. They found a significant association between FTO variants and BMI in depression cases, but not controls in two independent samples.^9^ In support of this, we find that BMI polygenic profile scores are more strongly associated with and explain more of the variance in BMI in depressed individuals compared with controls. This was also found in relation to GHQ and neuroticism. GHQ and neuroticism moderated the effect of BMI polygenic profile scores on BMI. Therefore, the effect of depression status on BMI-increasing alleles is not restricted to the FTO gene, but is observed across the spectrum of polygenic variation associated with BMI.

Psychological distress and depression are associated with disordered eating.^33^ In individuals at high genetic risk for obesity, the presence of mood disorders may have a greater impact on weight gain than those at low genetic risk. Cortisol levels are often increased in depressed individuals or those who have experienced stress. Elevated cortisol increases insulin secretion, which in turn promotes the accumulation of abdominal fat.^34^ Longitudinal studies have shown that baseline depression is associated with increased abdominal and visceral fat 5 years later.^35^ A recent study of 58 healthy women assessed for depression and previous-day stress were given two high-fat meals and found those who experienced the most stressors had the lowest postprandial resting energy expenditure.^36^ We find that in individuals with MDD or high GHQ scores, the effect of BMI-increasing alleles on BMI is increased. Further work is needed to understand the biological mechanisms that cause mood to influence BMI. However, these data suggest that increased BMI may be a response to endocrine fluctuations stimulated by current mood or lifetime depression, which then interacts with a genetic propensity to obesity.

There are a number of limitations to this study. The number of individuals in the reference MDD GWAS, which was used to create the polygenic risk scores, was low compared with the BMI GWAS (18 759 vs 123 865 total sample: 0 GWAS significant hits vs 19 GWAS significant hits). The power to detect association between the two disorders is biased towards the BMI polygenic profile scores. BMI polygenic profile scores are able to explain 4% of the variance in BMI, whereas MDD polygenic profile scores explain only 0.1% of the variance in MDD. We may have detected genetic overlap between BMI and MDD if the original GWAS for MDD were larger. Despite seeing a greater effect of BMI polygenic profile scores among depressed individuals, the amount of variance they explain is low, only 6.5% in this population. Another limitation is that previous studies have shown that the effect of BMI-increasing risk alleles is differential across MDD subtypes,^8^ and that atypical depression has a greater overlap with BMI. Atypical depression is characterized by hypersomnia and increased appetite and these cases typically present with a higher BMI than controls. In the present study, we did not restrict our analyses to obese or atypical cases as we chose to maximise our power to detect association in the available sample. Future studies including larger case–control samples would benefit from considering atypical depression status, although we note that the evidence suggesting that this is a valid and clinically useful stratification of depression is inconclusive.

Depression accounts for 8.2% of years lost to disability worldwide each year.^1^ Much of this disability is due to co-morbid physical conditions, which are partially explained by increased rates of overweight and obesity.^2^ We find that among individuals displaying current psychological distress or who have a lifetime diagnosis of MDD, BMI-increasing alleles have a stronger effect on BMI. Future studies replicating this finding are needed to confirm their significance, although this is the second study to show that MDD amplifies the effect of BMI-related genetic variants. By understanding the relationship between MDD and obesity we can better understand their mechanisms and develop interventions to reduce their considerable burden on those affected.

## Figures and Tables

**Table 1:** Demographic characteristics of GS:SFHS individuals in the current study

	*Total (13* *921)*	*Lifetime MDD (2030)*	*Controls (11* *836)*
Age (s.d.)	48.57 (15.07)	46.89 (12.97)^a^	48.88 (15.38)
Sex (% female)	59%	70.7%^a^	54.7%
BMI (s.d.)	26.87 (5.41)	27.61 (6.18)^a^	26.73 (5.26)
GHQ score (s.d.)	2.41 (4.06)	5.22 (6.21)^a^	1.91 (3.31)
Neuroticism (s.d.)	1.32 (0.77)	1.87 (0.61)^a^	1.23 (0.75)

Abbreviations: BMI, body mass index; GHQ, General Health Questionnaire; GS:SFHS, Generation Scotland: the Scottish Family Health Study; MDD, major depressive disorder.

a Significantly different from controls at *P*<2 × 10^−^^5^.

**Table 2:** Association between BMI polygenic profile scores and BMI, MDD status and GHQ at five different *P*-value threshold cutoffs

*BMI PGRS threshold*	*BMI*			*MDD*			*GHQ*			*Neuroticism*		
	β	r*^2^*	P*-value*	β	r*^2^*	P*-value*	β	r*^2^*	P*-value*	β	r*^2^*	P*-value*
*P*⩽0.01	0.17	0.034	**1.0 × 10^−^^93^**	0.001	0	0.71	0.029	0.0009	**0.0009**	−0.009	0	0.29
*P*⩽0.05	0.198	0.041	**7.2 × 10^−117^**	0.002	0	0.58	0.031	0.0009	**0.0004**	−0.001	0	0.91
*P*⩽0.1	0.2	0.042	**1.3 × 10^−^^118^**	0.004	0.0001	0.15	0.034	0.001	**0.0001**	0.004	0	0.66
*P*⩽0.5	0.197	0.041	**7.6 × 10**^−^**^115^**	0.003	0	0.38	0.032	0.001	**0.0002**	0.0005	0	0.96
*P*⩽1	0.18	0.035	**2.6 × 10^−^^100^**	0.002	0	0.45	0.026	0.0006	**0.0027**	−0.004	0	0.66

Abbreviations: BMI, body mass index; FDR, false discovery rate; GHQ, General Health Questionnaire; MDD, major depressive disorder; MDS, multidimensional scaling; PGRS, polygenic risk score.

Covariates include age, sex and four MDS components. Bolded *P*-values are significant after FDR correction.

**Table 3:** Interaction between BMI polygenic profile scores with MDD status, neuroticism and GHQ at five different *P*-value threshold cutoffs

*BMI PGRS threshold*	*MDD* × *PGRS* β *(s.e.)*	*MDD* × *PGRS* P*-value*	*Cur. DEP* × *PGRS* β *(s.e.)*	*Cur. DEP* × *PGRS* P*-value*	*GHQ* × *PGRS* β *(s.e.)*	*GHQ* × *PGRS* P*-value*	*Neurot* × *PGRS* β *(s.e.)*	*Neurot* × *PGRS* P*-value*
*P*⩽0.01	0.027 (0.02)	0.21	0.090 (0.04)	0.031	0.021 (0.008)	**0.009**	0.014 (0.008)	0.079
*P*⩽0.05	0.041 (0.02)	0.056	0.090 (0.04)	0.040	0.022 (0.008)	**0.005**	0.023 (0.008)	**0.004**
*P*⩽0.1	**0.064 (0.02)**	**0.0003**	0.085 (0.04)	0.051	0.023 (0.008)	**0.004**	0.023 (0.008)	**0.003**
*P*⩽0.5	0.037 (0.02)	0.086	0.018 (0.05)	0.68	0.024 (0.008)	**0.002**	0.018 (0.008)	0.020
*P*⩽1	0.021 (0.02)	0.33	−0.003 (0.04)	0.94	0.027 (0.008)	**0.0005**	0.016 (0.008)	0.048

Abbreviations: BMI, body mass index; Cur.Dep, current depression; FDR, false discovery rate; GHQ, General Health Questionnaire; MDD, major depressive disorder; PGRS, polygenic risk score.

In each case the dependent variable is BMI. Bolded *P*-values are significant after FDR correction.

## Notes

AMM has received financial support from Pfizer (formerly Wyeth), Janssen and Lilly. AMM has done consultancy work for Roche Pharmaceuticals. LJH has received financial support from Pfizer for work unrelated to the present study. The remaining authors declare no conflict of interest.
