# Supplementary material for: Major depressive disorder and current psychological distress moderate the effect of polygenic risk for obesity on body mass index
Source: Transl Psychiatry. 2015 Jun 30;5(6):e592–. doi: 10.1038/tp.2015.83 (PMC4490293; doi:10.1038/tp.2015.83)
Supplement: Supplementary Information [file tp201583x1.doc]

| **MDD PGRS threshold** | **BMI** | | | **MDD** | | | **GHQ** | | | **Neuroticism** | | |
| --- | --- | --- | --- | --- | --- | --- | --- | --- | --- | --- | --- | --- |
|  | **Beta** | **r2** | **p-value** | **Beta** | **r2** | **p-value** | **Beta** | **r2** | **p-value** | **Beta** | **r2** | **p-value** |
| **p ≤ 0.01** | 0.022 | 0.0004 | 0.01 | 0.002 | 0.000008 | 0.39 | 0.030 | 0.0008 | **0.0008** | 0.024 | 0.0004 | **0.005** |
| **p ≤ 0.05** | 0.011 | 0.0001 | 0.17 | 0.009 | 0.0006 | **0.003** | 0.039 | 0.0015 | **0.000009** | 0.036 | 0.001 | **0.00003** |
| **p ≤ 0.1*** | 0.013 | 0.0001 | 0.11 | 0.012 | 0.001 | **0.0001** | 0.037 | 0.0014 | **0.00001** | 0.033 | 0.0008 | **0.0002** |
| **p ≤ 0.5** | 0.011 | 0.0001 | 0.21 | 0.011 | 0.0009 | **0.0005** | 0.037 | 0.0013 | **0.00002** | 0.037 | 0.001 | **0.00003** |
| **p ≤ 1** | 0.010 | 0.00008 | 0.24 | 0.011 | 0.001 | **0.0002** | 0.038 | 0.0014 | **0.00002** | 0.038 | 0.001 | **0.00001** |

Supplementary Table 1) Association between MDD polygenic profile scores and BMI, MDD status and GHQ at 5 different p-value threshold cut-offs. Covariates include age, sex and 4 MDS components. Bold highlighted p-values significant after FDR-correction. *P-value threshold for MDD polygenic risk score which explains most of the variance in MDD.


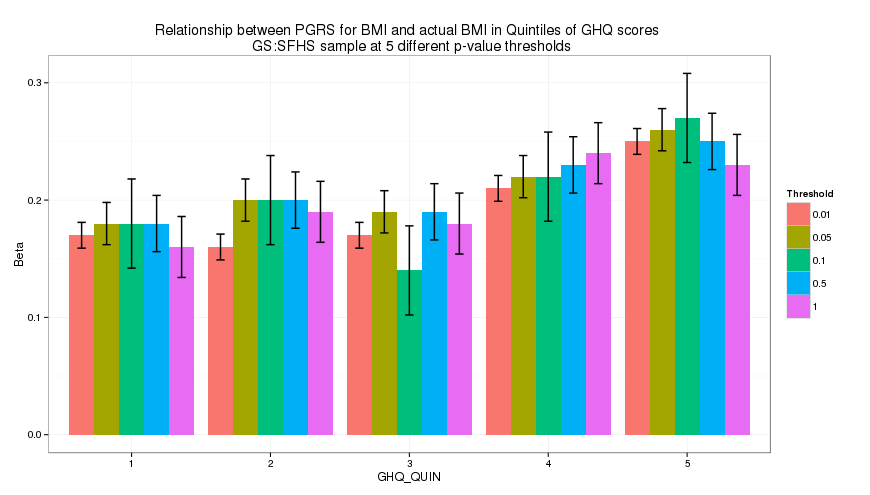


Supplemental Figure One) Relationship of BMI to BMI polygenic profile score in groups stratified by GHQ scores at each polygenic profile score p-value threshold. Group 1 contains individuals who scored 0 on the GHQ. Quartiles were created from individuals who scored greater than 1 on the GHQ. Group 2 contains individuals with a score of 1-2. Group 3 contains individuals with a score >2-3. Group 4 contains individuals who scored >3-6.5 and group 5 has those scoring higher than 6.5 on the GHQ. The y-axis represents the standardized beta co-efficient for the association between BMI and BMI polygenic risk in each of these quintiles. Error bars represent Standard Errors.
